# TUFM in health and disease: exploring its multifaceted roles

**DOI:** 10.3389/fimmu.2024.1424385

**Published:** 2024-05-29

**Authors:** Ning Liu, Bo Pang, Longfei Kang, Dongyun Li, Xia Jiang, Chuan-min Zhou

**Affiliations:** ^1^ The First Hospital of Hebei Medical University, Shijiazhuang, China; ^2^ Department of General Surgery, Hebei Key Laboratory of Colorectal Cancer Precision Diagnosis and Treatment, The First Hospital of Hebei Medical University, Shijiazhuang, China

**Keywords:** TUFM, mitochondria, virus, autophagy, cancer

## Abstract

The nuclear-encoded mitochondrial protein Tu translation elongation factor, mitochondrial (TUFM) is well-known for its role in mitochondrial protein translation. Originally discovered in yeast, TUFM demonstrates significant evolutionary conservation from prokaryotes to eukaryotes. Dysregulation of TUFM has been associated with mitochondrial disorders. Although early hypothesis suggests that TUFM is localized within mitochondria, recent studies identify its presence in the cytoplasm, with this subcellular distribution being linked to distinct functions of TUFM. Significantly, in addition to its established function in mitochondrial protein quality control, recent research indicates a broader involvement of TUFM in the regulation of programmed cell death processes (e.g., autophagy, apoptosis, necroptosis, and pyroptosis) and its diverse roles in viral infection, cancer, and other disease conditions. This review seeks to offer a current summary of TUFM’s biological functions and its complex regulatory mechanisms in human health and disease. Insight into these intricate pathways controlled by TUFM may lead to the potential development of targeted therapies for a range of human diseases.

## 1 Introduction to TUFM, a conserved gene from prokaryotes to eukaryotes

**Figure 1:**
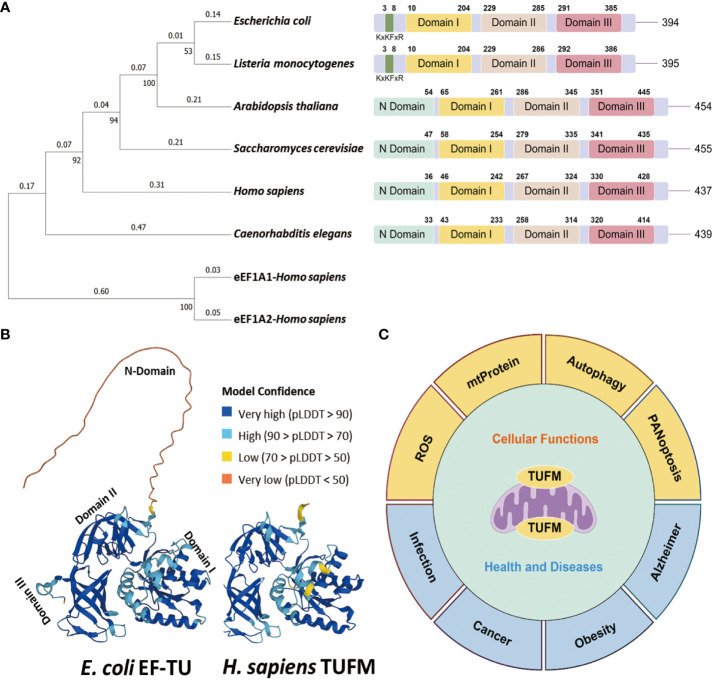
Conservation of TUFM protein. **(A)** Neighbor joining phylogenetic trees depicts the relationships among TUFM homologs (Left), and protein domain patterns of TUFM homologs juxtaposed with eEF1A (Right). **(B)** The AlphaFold 3D viewer showcases the structural comparison of TUFM proteins originated from *E*. *coli* (UniProt ID: P0CE47) and *H. sapiens* (UniProt ID: P49411) TUFM (8, 9). **(C)** Illustration of the diversified functions of TUFM, highlighting its multifaceted roles beyond mitochondrial protein translation and elongation.

Tu translation elongation factor, mitochondrial (TUFM, also known as P43; EF-TU; EFTU; COXPD4; EF-Tu MT) is a nuclear-encoded mitochondrial protein participating in mitochondrial polypeptide biosynthesis. Of note, TUFM is one of the most abundant proteins in the mitochondria and the bacterial cell (1–3). The initial exploration of TUFM rooted in the study of Yeast *Saccharomyces cerevisiae* in 1970 (4). In 1990s, the subsequent investigation involved the examination of TUFM expression pattern in both *Arabidopsis thaliana* and *Homo sapiens (* 5–7). TUFM proteins are conserved across a spectrum ranging from bacterial elongation factor Tu (EF-Tu) to eukaryotic TUFM homologs, and exhibit a higher degree of conservation in terms of structurally and functionally crucial amino acids (**Figures 1A, B**). *S. cerevisiae* TUFM and *Escherichia coli* EF-Tu are functionally interchangeable, showing notable nucleotide and amino acid homology, with percentages reaching 60% and 66%, respectively (4, 10, 11). In addition, distinctive structural variations are discernible among bacterial EF-Tu to eukaryotic TUFM homologs. The absence of the N terminus in bacterial EF-Tu distinguishes it from its eukaryotic counterparts TUFM, and bacterial EF-Tu possesses a distinctive KxKFxR motif (**Figures 1A, B**), serving as a pathogen-associated molecular pattern (PAMP) that elicits immune activation (12–15). A plausible scenario emerges wherein bacterial EF-Tu may be assimilated into host cells and undergo gradual evolution to assume the identity of TUFM. This evolutionary trajectory is marked by a functional shift from immune activation to immune suppression (14). Simultaneously, TUFM exhibits a significantly greater level of sequence divergence compared to its cytoplasmic counterparts, eukaryotic translation elongation factor 1 alpha (eEF1A) (**Figure 1A**), which is responsible for promoting recruitment of aminoacyl tRNAs to the ribosome (16, 17).

The TUFM gene is located on chromosome 16p11.2, accompanied by a putative pseudogene or variant TUFML in close proximity to the centromere of chromosome 17. TUFM encompasses three distinct protein domains including N-terminal GTP-binding domain (domain I), domain II, and a C-terminal domain (domain III) (**Figures 1A, B**). Notably, domain I incorporates a mitochondrion targeting sequence (MTS). Functionally, TUFM forms a ternary complex with GTP and mitochondrial aminoacyl-tRNAs (aa-tRNA), delivers the aa-tRNA to the A-site of the ribosome, and plays a critical role in mediating translation of mitochondrial genes, which has been covered in detail in many other articles (18–21). In bacteria, EF-Tu is predominantly phosphorylated at serine/threonine residues, and phosphorylation at these universally conserved residues inhibits protein synthesis by preventing ternary complex formation (22–24). In eukaryotic cells, TUFM has been identified as a potential target of Src Family Kinases Fyn and c-Src (25–27). TUFM-tyrosin-266 is one of the major c-Src phosphorylation targets, and phosphorylation at this site, as well as its phosphomimic mutation to a glutamate residue, inhibits ternary complex formation (27).

Beyond its fundamental role in mitochondrial protein translation and elongation (28, 29), TUFM has garnered increasing attention for its involvement in various physiological and pathological processes (**Figure 1C**). This paper provides a comprehensive review of the diverse roles of TUFM in both health and disease, with a specific emphasis on its contributions to viral infection and oncogenesis.

## 2 Diverse roles of TUFM in modulating autophagy

**Figure 2:**
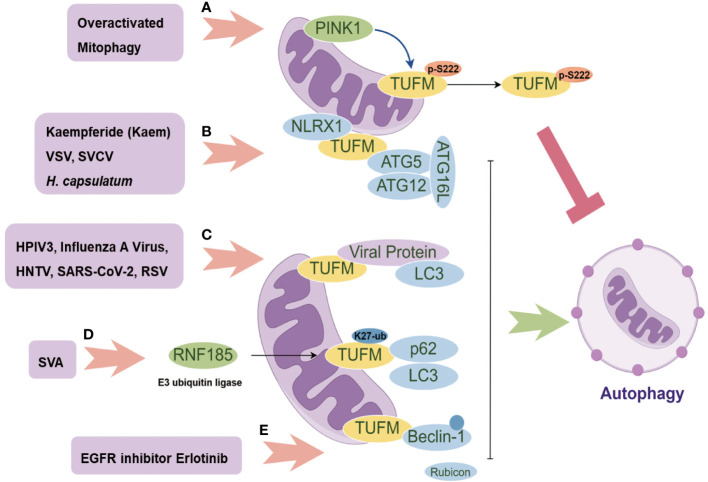
The Multifaceted Role of TUFM in Autophagy Modulation. PINK1 promotes the phosphorylation of TUFM at Serine222, leading to its delocalization from mitochondria to the cytoplasm and consequently releasing overactivated mitophagy **(A)**; The mitochondrial proteins NLRX1 and TUFM form a complex that recruits the autophagy E3-like complex ATG5-ATG12-ATG16L1, thereby inducing autophagy **(B)**; Several viral proteins can serve as autophagy adaptors, facilitating the interaction between TUFM and LC3 for autophagy induction **(C)**; SVA promotes the K27-linked ubiquitination of TUFM, which in turn enhances its interaction with p62 and LC3, mediating mitophagy **(D)**; Erlotinib promotes TUFM recruitment of Beclin-1 and its polyubiquitination, consequently disrupting its interaction with Rubicon for further autophagy induction **(E)**.

Mitophagy is a selective type of macro-autophagy by which dysfunctional or excessive mitochondria are sequestered by autophagic vesicles for degradation. In contrast to type I interferon (IFN) production, which normally restricts viral infection, the function of autophagy could be either the antiviral or proviral role depending on the viruses and types of host cells. Typically, mitophagy is an active process that protects the host against viral infection via degrading invading viral particles, enhancing antigen presentation, or boosting inflammatory and non-inflammatory responses (30–32). Conversely, mitophagy can sometimes be exploited for viral replication, and virus-induced mitophagy has been demonstrated to attenuate IFN responses. At present, the majority of studies on TUFM in mitophagy tend to concentrate on the subject of viruses (**Table 1**, **Figure 2**).

**Table 1:** Interaction between Viruses and TUFM.

Viruses	Viral Proteins	TUFM
Vesicular Stomatitis Virus (VSV)	–	Proviral (14, 33)
Human Parainfluenza Virus Type 3 (HPIV3)	M	Proviral (34)
Influenza A Virus rWSN PB2627E	PB2	Antiviral (35)
Hantaan virus (HNTV)	Gn	Proviral (36)
SARS-CoV-2	M	Unknown (37)
Spring Viremia of Carp Virus (SVCV)	–	Proviral (38)
Influenza A virus PR8	PB1-F2	Proviral (39)
African Swine Fever Virus (ASFV)	D1133L	Proviral (40)
Senecavirus A (SVA)	2C	Proviral (41)
Respiratory Syncytial Virus (RSV)	NS1	Proviral (42)
Japanese Encephalitis Virus (JEV)	NS3	Unknown (43)
Human Herpesvirus 8 (HHV-8)	vIRF-1	Antiviral (44, 45)

TUFM plays different roles in modulating mitophagy. On the one hand, TUFM interacts with autophagy E3-like complex ATG12-ATG5 and ATG16L1, thus serving as a tether recruiting NLR Family Member X1 (NLRX1) and promoting mitophagy (14, 33). Additionally, TUFM and NLRX1 are comparable in inhibiting Retinoic acid-inducible gene I (RIG-I)-like receptor (RLR) activation and promoting virus-mediated mitophagy that promotes vesicular stomatitis virus (VSV) infection (**Figure 2B**). Importantly, neither TUFM nor NLRX1 affect stimulator of interferon genes (STING, also known as MITA) pathway. TUFM proteins are conserved from fish to human (**Figure 1**), and mRNA levels of black carp TUFM (bcTUFM) are increased after spring viremia of carp virus (SVCV) infection (38). Similarly, bcTUFM cooperates with bcNLRX1 to inhibit mitochondrial antiviral-signaling protein (MAVS)-mediated antiviral signaling during SVCV infection (38) (**Figure 2B**). On the other hand, recent research has emphasized the precise modulation mechanism of TUFM in mitophagy, revealing that TUFM protein is a substrate of PINK1, which phosphorylates TUFM to suppress overactivated mitophagy (46). A PTEN-induced kinase 1 (PINK1)-dependent TUFM phosphoswitch at Serine222 determines conversion from activating to suppressing mitophagy. Mechanically, p-S222-TUFM is restricted predominantly to the cytosol, where it inhibits mitophagy by impeding ATG12-ATG5 formation (**Figure 2A**). Importantly, PINK1/TUFM is an evolutionarily conserved Parkin-independent pathway toward mitophagy.

Certain viral proteins induce complete mitophagy in the absence of Parkin, indicating the importance of TUFM in virus-induced mitophagy (**Figure 2C**), including influenza A virus PB1-F2, human parainfluenza virus (HPIV) M protein, Hantaan virus (HNTV) Gn protein, and respiratory syncytial virus (RSV) NS1 protein (34, 36, 39, 42). Substitution of glutamic acid (Glu, E) for lysine (Lys, K) at residue 627 of influenza A virus polymerase basic protein 2 (PB2) is regarded as overcoming host restriction and facilitate human infectivity (47). TUFM interacts with PB_262_7E as opposed to PB2_627_K (35, 48). By TUFM-dependent autophagy degradation for PB2_627_E, TUFM selectively restricts the replication of rWSN (a recombinant influenza A virus) PB2_627_E, while rWSN PB2_627_E induces TUFM-dependent autophagy. Conversely, TUFM is not involved in the replication of rWSN PB2_627_K or the autophagy that rWSN PB2_627_K induced (35). However, a recent study shows that TUFM benefits the replication of influenza A virus (39). Mechanically, influenza A virus PR8 PB1-F2 protein harbor a typical LIR motif, WxxL, at its C-terminal region and a TUFM interacting motif at its C terminal. The translocation of PB1-F2 to mitochondria is mediated by TUFM, and PB1-F2 in turn stimulates the interaction between TUFM and LC3, which mediates the induction of complete mitophagy. PB1-F2-induced mitophagy is critical for MAVS degradation and further type I IFN suppression.

Apart from influenza A virus, HPIV M protein (34), HTNV Gn protein (36), and RSV NS1 protein (42) translocate to mitochondria by interacting with TUFM, and then recruit LC3 to mitochondria for further TUFM and LC3 interaction (**Figure 2C**). Importantly, these interactions are required for mitophagy induction and further type I IFN inhibition. In addition, SARS-CoV-2 M protein interacts with TUFM, and autophagy is beneficial for SARS-CoV-2 replication (37) (**Figure 2C**), with unknown mechanism remained to be discovered. Notably, senecavirus A (SVA) 2C protein does not directly interact with LC3 under mitophagy induction status (41). SVA 2C protein promotes the K27-linked ubiquitination of TUFM catalyzed by the E3 ubiquitin ligase RNF185. Then, the ubiquitinated TUFM is recognized and bound by p62 (also called SQSTM1), which in turn interacts with LC3, thereby mediating complete mitophagy (**Figure 2D**). Of note, TUFM is discovered to interact directly with Beclin-1 and indirectly with the ATG12-ATG5 conjugate involved in mitophagosome formation (41).

African swine fever virus (ASFV) D1133 L and Japanese encephalitis virus (JEV) NS3 are both shown to interact with TUFM, while ASFV replication is considerably reduced by ectopic TUFM overexpression (40, 43). In addition, TUFM is involved in reactive oxygen species (ROS) inhibition (49, 50). Through a mechanism independent of ROS, NLRX1 facilitates LC3-Associated Phagocytosis (LAP) by interacting with TUFM which associates with autophagic proteins ATG5-ATG12 for LAPosome formation under fungus *histoplasma capsulatum* infection (**Figure 2B**). Of note, TUFM-mediated LAP induces MAPKs-AP-1 signal pathway activation for inflammatory cytokine response (51). In addition, Human Herpesvirus 8 (HHV-8) vIRF-1 is localized to mitochondria and stimulates TUFM dimerization for mitophagy induction which is associated with the inhibition of caspase-8-mediated apoptosis (44, 45) (**Table 1**).

## 3 TUFM’s role in cancer progression

**Figure 3:**
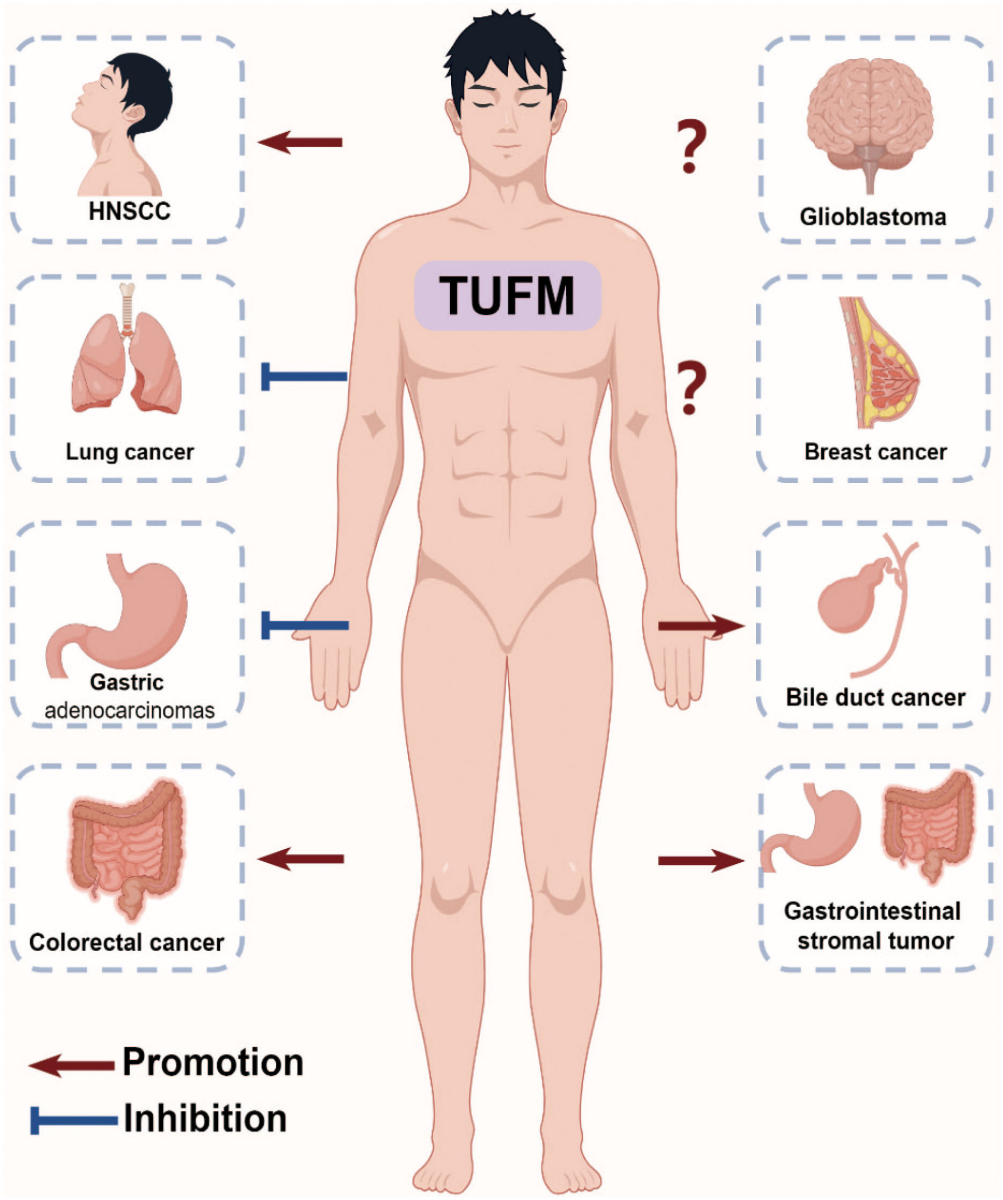
Role of TUFM in Cancer Progression. The multifunctional role of TUFM in various cancer progressions is evident, with overexpression in colorectal cancer leading to poorer clinical outcomes, promotion of proliferation and migration in gastrointestinal stromal tumors, negative correlations with serosal invasion and node involvement in gastric adenocarcinomas, promotion of autophagy and resistance to EGFR inhibitors in head and neck squamous cell carcinoma, inhibition of epithelial-mesenchymal transition in lung cancer, and controversial roles in glioblastoma and breast cancer.

Upregulation of TUFM has been reported in numerous types of tumors, including lung cancer (50), esophageal cancer (52), gastric cancer (53), intestinal cancer (54), bile duct cancer (55, 56), ovarian cancer (57, 58), pancreatic cancer (59), and others. Dysregulation of TUFM has been associated with various aspects of cancer progression, including tumor growth, metastasis, and therapeutic resistance (**Figure 3**).

### 3.1 Gastrointestinal cancer

Gastrointestinal cancer ranks top for incidence and second for mortality globally, constituting roughly 15.4% of all cancer cases (60). It has been well established that the majority of colorectal cancer (CRC) cases originate from adenomas (61, 62). Immunohistochemistry staining findings indicate a gradual increase in the expression of TUFM and tumor suppressor gene p53 from normal mucosa through adenoma to carcinoma tissues, correlating with the degree of dysplasia (63). Moreover, TUFM exhibits overexpression in CRC compared to matched normal colon mucosa, with minimal or weak expression in the stroma (54, 64). Increased TUFM expression in CRC correlates with poorer clinical outcomes, and serves as an independent prognostic factor for CRC patients. Specifically, TUFM expression significantly correlates with a higher 5-year recurrence rate in CRC patient, showing no significant association with additional clinical information, such as tumor node metastasis stage (54). TUFM is highly increased upon overexpression of microRNA-451a, a novel colorectal cancer-related gene (65). In addition, doxorubicin (DOX) and Berberine (BBR) have been shown to significantly suppress TUFM expression in CRC cells (66, 67). Mechanistically, TUFM is identified as a substrate of ubiquitin specific peptidase 5 (USP5). USP5 deubiquitinates TUFM at K48-linked chain and increases its stability, thereby promoting CRC cell growth, and TUFM, in turn, enhances the expression of Cyclin D1 (66). Consistent with the finding, both USP5 and TUFM expression are increased in CRC and are correlated with the prognosis of CRC patients.

Another *in vitro* analysis reveals that TUFM-knockdown results in decreased proliferation, as well as reduces lateral and vertical migration capacity of gastrointestinal stromal tumors (GISTs)-T1 and imatinib mesylate-resistant GIST-IR cells. In addition, there is an increase in the proportion of cells in the pre-G1 stage, with no alteration observed in the proportions of cells in the G_1_, S and G_2_/M stages in TUFM-knockdown GIST cells (68). In 5contrast to the reports mentioned above, high TUFM expression serves as a significant independent prognostic factor in gastric adenocarcinomas. TUFM is detected in both gastric adenocarcinoma and corresponding normal tissues, with high expression of TUFM also observed in gastric lymphocytes, vascular endothelial cells, and smooth muscle. Additionally, low expression of TUFM expression shows correlations with depth of serosal invasion and node involvement, and is associated with progression of gastric adenocarcinomas (53). Furthermore, TUFM expression varies among different gastric cancer cell lines, with high levels observed in SGC-7901, MKN-45, KatoIII, and AGS, while low levels are detected in MKN-28 and MKN-74 (53, 69).

### 3.2 Glioblastoma

Glioblastoma is the most common malignant primary brain tumor in adults, with the worst prognosis and overall survival approximately 15 months (70). TUFM has been proposed as a potential universal biomarker for glioblastoma (71–74). The protein levels of TUFM are found to be higher and comparable in glioblastoma tissue, neural stem cells (NSCs), glioblastoma stem cells (GSCs), and glioblastoma cell lines U251MG and U87MG, compared with normal brain tissue. Interestingly, the mRNA levels of TUFM are higher in GSCs, U251MG, and U87MG compared with normal brain tissue, glioblastoma tissue, and NSCs, of which TUFM mRNA expression does not exhibit significant differences between glioblastoma tissue and normal brain tissue (71). Significantly, the anti-TUFM nanobody Nb206 exhibits substantial cytotoxic effects, inducing apoptosis and necrosis, on the proliferation of GSCs and U87MG in particular, and to a lesser extent on U251MG (71). This finding is consistent with that genetic inhibition of TUFM-dependent mitochondrial translation suppresses GSCs proliferation (75). However, no significant reduction in cell growth is observed upon Nb206 treatment in NSCs, astrocytes, and HaCaT cells (71). These findings suggest that TUFM could play a significant role in promoting the progression and prognosis of glioblastoma. Although mRNA levels do not differ significantly between GBM and normal brain tissue, TUFM protein levels are higher in GBM tissue, indicating that post-transcriptional regulation of TUFM RNA could be a critical factor influencing its cancer-modulating function. Further studies are required to confirm these observations definitively.

### 3.3 Lung cancer

Lung cancer stands as a leading cause of cancer death worldwide, with approximately 2 million new cases and 1.7 million deaths annually (60). Recent research shows that TUFM protein levels exhibits a progressive decrease with the advancement of lung adenocarcinoma (50). Intriguingly, despite TUFM knockdown reducing tumor growth *in vivo*, it induces Epithelial-to-mesenchymal transition (EMT), as evidenced by decreased levels of epithelial adhesion marker proteins ZO-1 and E-cadherin, and increased protein levels of mesenchymal marker fibronectin. This finding aligns with the observation that the protein levels of TUFM are found to be decreased during TGF-β1-induced EMT and apoptosis (50, 76). Furthermore, TUFM knockdown diminishes mitochondrial respiratory chain activity, escalates glycolysis and ROS production, and enhances migration, invasion, and metastasis of lung cancer. Mechanistically, TUFM knockdown induces EMT through activation of the AMPK-GSK3β/β-catenin pathway (50). Notably, TUFM knockdown also promotes autophagy, as indicated by the conversion of LC3 in lung cancer (50), which contrasts with the classical role of TUFM in autophagy modulation (14).

### 3.4 Breast cancer

Breast cancer stands as the most commonly diagnosed cancer, with an estimated 2.3 million new cases and 6.8 million deaths annually (60). The current understanding of TUFM in breast cancer appears to be controversial. On one hand, TUFM knockdown induces EMT in MCF7 breast cancer cells, as evidenced by decreased levels of E-cadherin and γ-catenin (50). On the other hand, CBFB is found to interact with TUFM and enhances the binding of mitochondrial mRNAs to TUFM, thereby promoting breast cancer progression (77, 78). Interestingly, a novel resveratrol analog, HS-1793, exhibits anti-tumor activity in breast cancer by inhibiting the expression of mitochondrial translational protein TUFM and disrupting mitochondrial homeostasis, leading to sensitization of tumor cells to cell death specifically in MCF-7 cells (79). Similarly, ClpP agonists ONC201 and TR‐107 reduces the protein levels of TUFM and inhibits breast cancer progression (80–83).

### 3.5 Head and Neck Squamous Cell Carcinoma

Current studies show that blocking epidermal growth factor receptor (EGFR) inhibits HNSCC initiation and maintenance (84), with the inhibition of autophagy sensitizing HNSCC to EGFR blockade (85). Regarding TUFM, a reduction in its expression is associated with a poor response to EGFR inhibitor cetuximab or gefitinib treatment (86). Mechanistically, the deficiency of either NLRX1 or TUFM results in impaired autophagy when treated with EGFR inhibitors in HNSCC cells. The NLRX1/TUFM complex has been identified as a novel anchorage site for autophagy, facilitating the recruitment of Beclin-1 to mitochondria, enhancing its polyubiquitination, and disrupting its interaction with Rubicon (**Figure 2E**). Although no direct interaction between NLRX1 and Beclin-1 is observed, it is noteworthy that this protein complex also plays a crucial role in facilitating unfolded protein response (UPR) activation, potentially serving as an additional mechanism to enhance autophagy (86).

## 4 Alzheimer’s disease

Recent studies have shown a reduction in TUFM protein levels in the brains of individuals with Alzheimer’s disease, as well as in mouse models of such disease (87–89). The downregulation of TUFM has been associated with decreased levels of β-amyloid converting enzyme 1 (BACE1) and its catalytic product, β-amyloid protein (87, 90). Silencing TUFM results in enhanced RNA stability of BACE1 through ROS but not NF-κB-dependent translation modulation. Furthermore, silencing TUFM is linked to apoptosis induction and tau phosphorylation, both of which are mitigated by the mitochondria-targeted antioxidant TEMPO. In addition, Kaempferide (Kaem), a natural flavonoid compound, is shown to facilitate the autophagic clearance of microtubule-associated protein tau by targeting TUFM (91). Taken together, these findings indicate that targeting TUFM may hold promise as a therapeutic strategy for ameliorating Alzheimer’s disease.

## 5 Obesity

The relationship between TUFM-dependent autophagy and obesity, as well as its impact on the insulin cascade, has been extensively documented in the literature (92–96). In rats subjected to high-fat diet, TUFM expression is found to be increased in adipose tissue, liver and extensor digitorum longus muscle (97). Furthermore, pediatric adipose tissue samples from individuals with obesity also exhibits a significant upregulation of TUFM (98). Mouse models have further elucidated the effects of diet and physical activity on TUFM expression, showing that a western diet and sedentary lifestyle can lead to decreased TUFM expression in muscles compared to a normal chow diet and voluntary wheel running (99). Kaem exhibits a high binding affinity to the TUFM G172 and K256 residues, facilitating its interaction with the ATG12-ATG5 complex to induce autophagy and promote lipid degradation (49) (**Figure 2B**). These results offer valuable insights into the potential therapeutic implications of targeting TUFM in the management of metabolic syndrome.

## 6 Cardiovascular diseases

TUFM is significantly upregulated in individuals diagnosed with pulmonary arterial hypertension (PAH) (100, 101), with predominant expression observed in pulmonary arterial smooth muscle cells (PASMCs) and minimal to absent expression in endothelial cells in a rat model of monocrotaline-induced PAH. Silencing of TUFM is shown to mitigate monocrotaline-induced PAH by enhancing apoptosis and reducing mitophagy (101). Mechanically, TUFM is found to promote PASMC proliferation and is induced under hypoxic conditions in PASMCs (101, 102). Additionally, TUFM silencing alleviates mitophagy and enhances apoptosis in PASMCs exposed to hypoxia, which is associated with AMPK/mTOR Pathway (101).

TUFM is widely expressed in the heart (103). In AC-16 cardiomyocytes, FUNDC1 recruits TUFM directly and facilitates its translocation to the mitochondria. This interaction between FUNDC1 and TUFM plays a crucial role in maintaining mitochondrial DNA stability and preventing PANoptosis-associated cell death when exposed to DOX (104). Additionally, the depletion of TUFM results in similar effects on the release of mitochondrial DNA and the upregulation of PANoptosis. It is important to note that the administration of DOX leads to a decrease in TUFM expression in heart tissues, a phenomenon that is not influenced by FUNDC1. In mouse models of diabetes and diabetic cardiomyopathy, mitochondrial argonaute 2 (ago2) is found to directly interact with TUFM to facilitate the translation of electron transport chain subunit, consequently leading to a decrease in ROS and providing protection (52). Of note, Ago2 is observed to enhance the binding of TUFM to mitochondrial mRNAs, while TUFM does not impact the interaction between Ago2 and mitochondrial RNAs. Furthermore, the increase in mitochondrial-encoded subunits, specifically cytochrome b (CYTB), targed by miRNAs miR-133a-3p, miR-143-3p, and miR-21, is lost in Ago2 or TUFM deficient myocytes.

## 7 Non-alcoholic steatohepatitis

A mitochondrial Mortality factor 4-like protein 1 (MRG15)-TUFM axis is tightly involved in the progression from simple steatosis to NASH (105). Reduced levels of TUFM are observed in the livers of patients with NASH or mouse models of NASH, with a negative correlation with MRG15 expression. Intriguingly, no significant changes are found in TUFM mRNA levels in liver tissues or primary hepatocytes. Mechanistically, MRG15 interacts with and deacetylates TUFM, specifically at the K82 and K91 sites within mitochondria. This interaction facilitates TUFM degradation through the mitochondrial ClpXP protease system, ultimately resulting in impaired mitophagy, increased ROS, and activated NLRP3 inflammasome.

## 8 Summary and further directions

TUFM, initially identified as a nuclear-encoded mitochondrial protein in yeast, exhibits high conservation from prokaryotes to eukaryotes (4–7), yet the evolutionary functions and origins of TUFM remain largely elusive. Although recognized for its involvement in mitochondrial protein translation, recent studies have unveiled its multifaceted functions beyond this realm. TUFM not only regulates programmed cell death but also shows potential as a drug target for treating virus- and cancer-related diseases. Mutations in TUFM have been linked to an early fatal outcome and a range of diseases, such as multiple sclerosis (106), dysplastic leukoencephalopathy (107), and premature ovarian insufficiency (108).

It is of significance to note that TUFM displays a higher susceptibility to proteinase K digestion in comparison to mitochondrial inner and matrix proteins, yet is similar to outer membrane proteins, indicating its steady-state presence on the mitochondrial outer membrane (44, 46). This finding may provide insight into the potential interaction between TUFM and cytosolic viral or host proteins for further mitophagy induction. Furthermore, prior research has indicated that Influenza A PB1F2 localizes to the mitochondrial inner membrane space, potentially facilitating its interaction with TUFM (39, 109). Based on our knowledge, TUFM is a dual-localized protein with a mitochondrial targeting sequence (46). It translocates to the matrix side of mitochondria through the outer and inner mitochondrial membrane channel under resting states. However, when overactivated mitophagy occurs, phosphorylation of TUFM at Ser222 by PINK1 switches its role from activating to suppressing mitophagy (46). This phosphorylated form of TUFM is mainly found in the cytosol, potentially protecting mitochondria from excessive degradation. The regulation of TUFM activation appears to be tightly controlled at spatially and temporally levels, although the specific mechanisms remain incompletely understood.

Recent research has elucidated the involvement of TUFM in non-canonical mitophagy during viral infection, where viral proteins exploit TUFM for mitophagy induction and viral replication. An intriguing area for further investigation lies in elucidating the specific mechanisms underlying TUFM-dependent autophagy. Additionally, in the context of cancer, increased expression of TUFM may play a role in promoting tumor metastasis and progression. Exploring the molecular pathways through which TUFM contributes to the initiation, progression, and resistance to therapy in cancer within the tumor microenvironment may reveal novel biomarkers and therapeutic targets for the treatment of cancer. Additionally, the evaluation of small molecule compounds or anti-TUFM antibody-drug conjugates could be a viable strategy to modulate TUFM activity for the management of diverse diseases.

In summary, continued investigation into the diverse functions of TUFM in both health and disease is imperative for elucidating its therapeutic efficacy and devising precise interventions for a range of human diseases.

## Author contributions

NL: Writing – original draft. BP: Writing – original draft. LK: Writing – review & editing. DL: Writing – review & editing. XJ: Supervision, Writing – review & editing. C-MZ: Conceptualization, Supervision, Writing – review & editing.
